# Environmental Uncertainty, Environmental Regulation and Enterprises’ Green Technological Innovation

**DOI:** 10.3390/ijerph19169781

**Published:** 2022-08-09

**Authors:** Jinyong Chen, Xiaochi Wang, Wan Shen, Yanyan Tan, Liviu Marian Matac, Sarminah Samad

**Affiliations:** 1 Business School, Hubei University, Wuhan 430000, China; 2 Accounting School, Zhongnan University of Economics and Law, Wuhan 430073, China; 3 Department of Accounting and Audit, Bucharest University of Economic Studies, 010374 Bucharest, Romania; 4 Department of Business Administration, College of Business and Administration, Princess Nourah bint Abdulrahman University, Riyadh 11671, Saudi Arabia

**Keywords:** environmental uncertainty, environmental regulation, green technological innovation

## Abstract

This paper examines the impact of environmental uncertainty and environmental regulation on enterprises’ green technological innovation, using a panel data of Chinese A-share listed companies in Shanghai and Shenzhen from 2005 to 2019 to conduct an empirical study using an OLS model and Poisson regression model. We employ environmental complexity and environmental dynamism to measure environmental uncertainty, and we have the following findings: first, both environmental uncertainty and environmental regulation promote enterprises’ green technological innovation, while environmental regulation has positive moderating effects on the relationship between environmental uncertainty and enterprises’ green technological innovation; second, environmental complexity positively affects enterprises’ green technological innovation, while environmental dynamism has negative effects on enterprises’ green technological innovation; third, environmental regulation accentuates the relationship between environmental complexity and green technological innovation, while it weakens the relationship between environmental dynamism and green technological innovation.

## 1. Introduction

In recent years, climate change and environmental sustainability have become some of the most pressing global economic issues. In the context of globalization and integration, all countries are actively exploring the future path of sustainable development. At present, China is in a stage of transition from high-speed economic growth to high-quality development. The white paper “China’s Energy Development in a New Era” released by the Information Office of the State Council of China in December 2020 pointed out that the problems of resource and energy waste and environment pollution brought by original extensive development are increasingly prominent. The 19th National Congress stressed that “establishing and perfecting an economic system with green and low-carbon circular development” is the only way to achieve high-quality development, which means that we should revitalize the economy, making it innovative and competitive through green development; thus, pursing new economic growth through green technological innovation is inevitable. China’s Communist Party’s “14th Five-Year Plan” puts forward a two-carbon strategy of “striving to reach a carbon peak by 2030 and achieving carbon neutrality by 2060” to promote green development to a new height. The Peak Carbon Dioxide Emissions Action Plan for the Period up to 2030, released on 26 October 2021, set higher requirements for carbon dioxide emissions per unit of gross domestic product and the proportion of non-fossil energy consumption in China in 2025 for the current and next five-year plans, which lays a solid foundation for the realization of peak carbon dioxide emissions. However, China is a developing country and has a long way to go in industrialization and urbanization, and high-quality development and the improvement of people’s well-being are still primary goals, which means that the rigid demand for energy in China will not decrease in the short term. Peak carbon dioxide emissions are a top priority in the “decoupling” of high-quality economic development from carbon dioxide emissions. In other words, economic development is no longer at the price of carbon emissions, which marks a country’s or region’s transition to a green and low-carbon economy and transition to high-quality economic development.

As an important participant in the market economy, enterprises are facing many of problems, such as increasing environmental pollution, Russia–Ukraine conflict and Sino–US trade friction. The increased uncertainty of the external environment implies more risks taken by enterprises. Therefore, enterprises must change production methods through green technological innovation to improve production efficiency and enhance market competitiveness. At the same time, enterprises should abide by and make use of the environmental policies issued by the government to maximize the utilization of resources and establish a green technological innovation system, thus accelerating the transformation to a green economy. In view of this background, this paper employs two dimensions to illustrate environmental uncertainty, which include environmental dynamism and environmental complexity. Next, we discuss the impact of environmental uncertainty on enterprises’ green technological innovation from the overall uncertainty and the two dimensions, respectively. We further investigate the moderating effect of environmental regulation on the relationship between environmental uncertainty and enterprises’ green technological innovation. On one hand, it expands the research on innovation motivation. On the other hand, it is of great significance in guiding enterprises to carry out green innovation activities and has important implications on government policies.

The contributions of this paper are as follows: first, we decompose environmental uncertainty into environmental dynamism and environmental complexity and then study their effects on enterprises’ green technological innovation, which enriches the motivations of innovation; second, we present some new findings. Although environmental uncertainty can stimulate enterprises’ green technological innovation as a whole, environmental complexity and environmental dynamism have different impacts on enterprises’ green technological innovation. Additionally, we have confirmed the moderating effect of environmental regulation on environmental uncertainty and enterprises’ green technological innovation, which expands the research on external environment, macro-policies, and enterprises’ green technological innovation, thus providing a theoretical basis and empirical support for stakeholders.

## 2. Theoretical Background and Research Hypothesis

### 2.1. Environmental Uncertainty and Enterprises’ Green Technological Innovation

High environmental uncertainty will affect the choice of future development strategy of enterprises, which may not only induce operating risks and financial risks, but also hinder endogenous financing. The management will adopt a conservative strategy, appropriately reduce the scale of research and development activities of corporations and innovate more cautiously [1]. Especially for heavily polluting industries, due to their large emissions and serious pollution, they will undoubtedly face more stringent environmental policies. However, innovation requires a rather long cycle and high investment, which restrains the motivation of heavily polluted industries [2,3,4]. Corporations generally face macro-level and micro-level uncertainties, which usually include macro-level effects such as economic cycle fluctuations, policy changes and market competition [5,6], and micro-level factors such as changes in capital structure, stock price fluctuations and sales fluctuations [7,8]. This paper measures the environmental uncertainty by complexity and dynamism of the environment. Complexity is the degree of fierce competition in the external environment. From the perspective of enterprise, the green technological innovation of enterprises is the main way to develop their own core competitiveness. It can design distinctive products, prevent product homogenization and enhance the market competitiveness of enterprises. From the perspective of the market, the public’s awareness of green products is gradually strengthening, requiring enterprises to produce products that are in line with public interests and environmentally friendly [9,10]. Therefore, when industry competition intensifies, inefficient enterprises will be eliminated, and high-quality enterprises that actively innovate and improve production efficiency will survive, so that the resource utilization efficiency and technological innovation level of the whole industry will also be improved [11].

As one of the manifestations of environmental uncertainty, environmental dynamism is the change in and unpredictability of the environment, which represent frequent market fluctuations. With the fluctuation of earnings and stock price, it is difficult for enterprises to estimate earnings and market value, which may ultimately affect the innovation decision making of enterprises [12]. Enterprises that can maintain their leading position in the industry and have core competitive advantages rely on rare resources that are difficult to copy, obtain and imitate, and these resources must be generated within enterprises. Changes in the external environment not only cause enterprises to lose scarce resources but also increase the cost of obtaining resources from the outside, which, in turn, may damage the original innovation ability of enterprises and hinder enterprises’ further technological innovation. However, in the long run, the external environment faced by the enterprises is always changing. If enterprises want to achieve profit maximization, they must carry out green technological innovation, reduce their production and operation costs, change their production mode, improve the utilization rate of resources, and realize the maximization of their enterprise value. Based on the above analysis, Hypothesis 1 is proposed.

**H1a.**  *On the whole, environmental uncertainty promotes enterprises’ green technological innovation.*

**H1b.**  *Environmental complexity promotes enterprises’ green technological innovation.*

**H1c.**  *Environmental dynamism inhibits enterprises’ green technological innovation.*

### 2.2. Environmental Regulation and Enterprises’ Green Technological

At present, the environment is of great concern to the whole world. As one of the plans to improve the people’s livelihood, environmental protection must be started from the original source and transformed from the end of management to the forefront. Therefore, improving enterprises’ technological innovation level is the best approach to achieve energy conservation and emission reduction and social sustainable development [13]. However, innovation usually has the disadvantages of high investment and long return period, which make enterprises flinch. Therefore, the enthusiasm for green technological innovation is not high. Generally, passive innovation is preferred over active change. At this time, the “visible hand” of the government and the “invisible hand” of the market need to play a guiding and motivating role to stimulate enterprises’ initiative in technological innovation [14].

Due to the negative externalities of environmental pollution, the market mechanism cannot restrict enterprises’ environmental pollution behavior, and enterprises’ behavior must be regulated by the government. The government imposes mandatory pressure on enterprises through order-type environmental regulations, forcing them to innovate passively; market-driven environmental regulations incentivize enterprises through innovative subsidies, environmental tax relief and technical support. At the same time, the public’s awareness of protecting the environment has been improved. They will take the initiative to supervise and report the pollution discharge behavior of enterprises and put an end to the high pollution and high energy consumption business model of enterprises. Therefore, in order to achieve long-term economic benefits, enterprises must adapt to the green development trend and take the initiative to carry out green technological innovation.

Existing theoretical and empirical studies indicate that environmental regulation can promote enterprises’ green technological innovation. Villegas and Coria (2010) have studied whether there are differences in their role in promoting technological innovation in enterprises by specifying environmental regulations of sewage taxes and trading permits [15]. Ouyang et al. (2020) and Tian et al. (2021) stressed that the process of China’s environmental governance has reached a critical moment. Strictly implementing environmental policies should not be relaxed, and efforts should be made to increase support for enterprises to ensure that they are more motivated to innovate in green technologies [16,17]. Peng et al. (2021) found the logical chain of “environmental regulation—stimulating green innovation intention—promoting green innovation behavior” and found that industrial agglomeration plays an active moderating role in the logical chain [18]. Fullerton and Metcalf (2001) divided environmental regulations into command-and-control environmental regulation tools and incentive environmental regulation tools [19]. Wang et al. (2020) empirically test whether the command-and-control environmental regulation policy implemented by the Chinese government has a positive impact on green technological innovation [20]. Based on the above analysis, Hypothesis 2 is proposed.

**H2.**  *Environmental regulation helps to promote enterprises’ technological Innovation*.

### 2.3. Environmental Uncertainty, Environmental Regulation and Enterprises’ Green Technological Innovation

Cai et al. (2020) found that due to industry heterogeneity, the impact of environmental regulations on technological innovation in different industries is different [21]. European and American carbon emission trading schemes allow enterprises to fulfill their obligation of reducing pollution by purchasing quotas from other emitting countries, which may reduce their motivation to innovate because of the uncertainty in the innovation process [22,23]. Porter and Van der Linde (1995) proposed that strict and appropriate environmental regulation will promote enterprises’ technological innovation, and under dynamic conditions, it can create a win–win situation, improving environmental quality and the productivity and competitiveness of manufacturers [24]. It can be seen that when enterprises are faced with the uncertainty of fierce external competition, under the supervision and encouragement of environmental regulations, enterprises will favor green technological innovation, research and develop new green products to meet public demand, increase industry entry barriers and consolidate their market position. Many scholars also think that environmental regulation has a negative impact on enterprises’ technological innovation [25,26]. Walley and Whitehead (1994) put forward the view that even if the government promotes enterprises’ innovation by widening financing channels and other ways, innovation will be exclusive under the condition of limited funds [27]. The compliance cost hypothesis points out that strict environmental regulation implies additional costs for compliant firms, thus crowding out capital investments that could otherwise be used for innovation [28]. Facing the fluctuation of the market, it is very difficult for enterprises to obtain the funds needed for innovation from external investors, and due to the increasingly strict external environmental policies, enterprises have to put out part of the funds to pay for environmental pollution, which will make enterprises more resistant to green technological innovation. Based on the above analysis, Hypothesis 3 is proposed:

**H3a.**  *On the whole, environmental regulations accentuate the impact of environmental uncertainty on enterprises’ green technological innovation.*

**H3b.**  *Environmental regulation has a positive moderating effect on environmental complexity and enterprises’ green technological innovation.*

**H3c.**  *Environmental regulation has a negative moderating effect on environmental dynamism and enterprises’ green technological innovation.*

## 3. Research Methodology

### 3.1. Research Samples and Data Sources

This paper uses the data of A-share listed companies in Shanghai and Shenzhen from 2005 to 2019 and employs STATA16.0 (StataCorp LLC: College Station, TX, USA) and Excel to process the data. The data of green technological innovation are from the State Intellectual Property Office. The environmental regulation data are mainly from China Environmental Statistics Yearbook; other research data are mainly from CSMAR database. We delete the samples with names of ST, from financial industry and whose key information is false or missing. We finally obtained 21,359 observations. Moreover, in order to prevent the empirical results from being biased, Winsorize is applied to the continuous variables in the upper and lower 1% percentile.

### 3.2. Variables

#### 3.2.1. Dependent Variable

Based on the classification catalogue of environmentally friendly patent technologies, this paper classifies the patents in this catalogue as green patents. Taking Zhao et al. [29] as reference, we measure enterprises’ green technological innovation by taking the natural logarithm of the sum of green invention patents and utility model patents in sample companies.

#### 3.2.2. Explanatory Variable

##### Environmental Uncertainty

Environmental uncertainty (*EU*) is measured by the interaction of environmental complexity and dynamism, namely *HHI* × *EU_adj*

##### Environmental Complexity

The Herfindahl–Hirschman Index is used to measure the complexity of environmental uncertainty, and the specific calculation is shown in Model (1)

(1) $$HHI_{t}={\sum_{i=1}^{n}\left(\frac{X_{it}}{X_{t}}\right)}^{2}$$

Among them, *X_t_* is the total revenue from main business of the industry to which the enterprise belongs in year *t*, *X_it_* is the total revenue from main business of company *i* in year *t*, and *X_it_*/*X_t_* is the market share of the industry accounted for by company *i* in year *t*. *HHI* value changes inversely with the intensity of industry competition. Therefore, in this paper, the Herfindahl–Hirschman Index is negatively treated as an index to measure industry competition. The greater its value, the greater the intensity of industry competition.

##### Environmental Dynamism

Referring to the research method of scholar Ghosh and Olsen [7], the ordinary least square method is used to regress the operating income data of the past five years, and the residual error is the abnormal sales income. Model (2) presents the details. Then, the standard deviation of the abnormal sales income in the past five years is divided by the average value to obtain the environmental dynamism without industry adjustment. Finally, the index of dynamism of environmental uncertainty after adjustment is calculated by dividing by the industry median value.

(2) $$Sale=\phi_{0}+\phi_{1}Year+\varepsilon$$

Among them, *Sale* is sales revenue, and *Year* is the annual variable.

#### 3.2.3. Moderator Variable

We take Environmental regulation (*ER*) as a moderator variable. Different from a single indicator, with reference to the research method of Zhao and Sun [30], we measure the provincial environmental regulation intensity by industrial sulfur dioxide, wastewater discharge and smoke discharge per unit output value. The specific steps are as follows.

##### Standardize Pollutant Discharge

See Model (3) for calculation method

(3) $${E^{s}}_{ij}=\frac{E_{ij}-\min E_{j}}{\max E_{j}-\min E_{j}}$$

Since industrial sulfur dioxide, wastewater and soot emissions are measured differently, the indicators are standardized through Model (3) to reduce the effect of magnitude on the composite index. Among them, *E_ij_* is the discharge of the *j*-type pollutant in Province *i*, Max (*E_j_*) and Min (*E_j_*) are the maximum and minimum values of indicator j in Province *i*, respectively, and *E^s^_ij_* is the normalized value

##### Adjust the Pollution Coefficient

As the proportion of pollution discharge varies greatly among different provinces, adjusting the pollution coefficient can reflect the pollution degree of each province more accurately. The calculation formula is shown in Model (4)

(4) $$W_{j}=E_{ij}/{\bar{E}}_{ij}$$

*W_j_* represents the pollution coefficient, and *E_ij_* represents the average value of the emissions of pollutant *j*. Definition of Main Variables is shown in Table 1.

##### Calculate the Provincial Environmental Regulation Intensity *ERS_i_*

After the standardization of the indicators and calculation of the weights, the weighting method is used to integrate the index synthetically. The calculation formula is shown in Model (5)

(5) $$ERS_{i}=\frac{1}{3}\sum_{j}^{3}W_{j}E_{ij}^{s}$$

### 3.3. Model Setting

#### 3.3.1. Model of Direct Effect of Environmental Uncertainty on Enterprises’ Green Technological Innovation

Model (6) examines the overall effect of environmental uncertainty on enterprises’ green technological innovation. Model (7) studies the effect of the environmental complexity on enterprises’ green technological innovation by Herfindahl–Hirschman index. Model (8) tests the effect of environmental dynamism on enterprises’ green technological innovation by industry-adjusted sales revenue fluctuation

(6) $$\begin{array}{l} \ln GI=\alpha_{0}+\beta_{1}EU+\beta_{2}S\mathrm{ize}+\beta_{3}L\arg est+\beta_{4}Lev+\beta_{5}CGB\\ +\beta_{6}TobinQ+\beta_{7}ROA+\beta_{8}Dud+\beta_{9}Dual+\varepsilon \end{array}$$

(7) $$\begin{array}{l} \ln GI=\alpha_{0}+\beta_{1}HHI+\beta_{2}S\mathrm{ize}+\beta_{3}L\arg est+\beta_{4}Lev+\beta_{5}CGB\\ +\beta_{6}TobinQ+\beta_{7}ROA+\beta_{8}Dud+\beta_{9}Dual+\varepsilon \end{array}$$

(8) $$\begin{array}{l} \ln GI=\alpha_{0}+\beta_{1}EU\_adj+\beta_{2}S\mathrm{ize}+\beta_{3}L\arg est+\beta_{4}Lev\\ +\beta_{5}CGB+\beta_{6}TobinQ+\beta_{7}ROA+\beta_{8}Dud+\beta_{9}Dual+\varepsilon \end{array}$$

#### 3.3.2. Model of Effect of Environmental Regulation on Enterprises’ Green Technological Innovation

Model (9) tests the impact of environmental regulations on enterprises’ green technological innovation by the least square method (OLS).

(9) $$\begin{array}{l} \ln GI=\alpha_{0}+\beta_{1}ER+\beta_{2}S\mathrm{ize}+\beta_{3}L\arg est+\beta_{4}Lev+\beta_{5}CGB\\ +\beta_{6}TobinQ+\beta_{7}ROA+\beta_{8}Dud+\beta_{9}Dual+\varepsilon \end{array}$$

#### 3.3.3. Environmental Uncertainty, Environmental Regulation and Enterprises’ Green Technological Innovation

In order to verify the moderating effect of environmental regulation on environmental uncertainty and green technological innovation, the interaction term *EU* × *ER* between environmental uncertainty and environmental regulation is added, as shown in Model (10); in order to verify the moderating effect of environmental regulation on environmental complexity and green technological innovation, the interaction term *HHI* × *ER* between environmental complexity and enterprises’ green technological innovation is added (see Model (11)); in order to verify the moderating effect of environmental regulation on environmental dynamism and green technological innovation, the interaction term *EU_adj* × *ER* between environmental dynamism and enterprises’ green technological innovation is added (see Model (12)).

(10) $$\begin{array}{l} \ln GI=\alpha_{0}+\beta_{1}EU+\beta_{2}ER+\beta_{3}EU*ER+\beta_{4}S\mathrm{ize}+\beta_{5}L\arg est\\ +\beta_{6}Lev+\beta_{7}CGB+\beta_{8}TobinQ+\beta_{9}ROA+\beta_{10}Dud+\beta_{11}Dual+\varepsilon \end{array}$$

(11) $$\begin{array}{l} \ln GI=\alpha_{0}+\beta_{1}ER+\beta_{2}HHI+\beta_{3}ER*HHI+\beta_{4}S\mathrm{ize}+\beta_{5}L\arg est\\ +\beta_{6}Lev+\beta_{7}CGB+\beta_{8}TobinQ+\beta_{9}ROA+\beta_{10}Dud+\beta_{11}Dual+\varepsilon \end{array}$$

(12) $$\begin{array}{l} \ln GI=\alpha_{0}+\beta_{1}ER+\beta_{2}EU\_\mathrm{adj}+\beta_{3}ER*EU\_\mathrm{adj}+\beta_{4}S\mathrm{ize}\\ +\beta_{5}L\arg est+\beta_{6}Lev+\beta_{7}CGB+\beta_{8}TobinQ+\beta_{9}ROA+\beta_{10}Dud\\ +\beta_{11}Dual+\varepsilon \end{array}$$

## 4. Results

### 4.1. Descriptive Statistics

Table 2 is a descriptive statistical result of each variable. We find that the median of enterprises’ green technological innovation (ln*GI*) is 0, the average value is 0.398, the maximum value is 3.850, and the standard deviation is 0.831. Additionally, the overall level of green technological innovation in our country is low, and the difference is not significant as a whole. The minimum environmental uncertainty (*EU*) is −1.470, the average value is −0.171, and the standard deviation is 0.238, indicating that the uncertainty of the external environment experienced by the enterprise is different. The average value of environmental complexity (*HHI*) is −0.134, the minimum value is −0.793, the maximum value is −0.02, the average value of environmental dynamism (*EU_adj*) is 1.287, the minimum value is 0.13, the maximum value is 6.705, and the standard deviation is 1.144, which shows that there are differences in the degree of environmental fluctuation that each enterprise should deal with. The average value of the environmental regulation (*ER*) is 0.693, the minimum value is 0, the maximum value is 2.179, and the standard deviation is 0.608. It can be concluded that the external environmental policy pressures on various types of enterprises are different.

### 4.2. Correlation Analysis

The correlation analysis results of each variable in this paper are shown in Table 3. The correlation coefficient between environmental uncertainty (*EU*) and green technological innovation (ln*GI*) is 0.07, which indicates that the *EU* has a positive impact on enterprises’ green innovation activities. The correlation coefficients of environmental complexity (*HHI*), environmental dynamism (*EU_adj*) and green technological innovation (ln*GI*) are 0.02 and −0.08, respectively. It can be preliminarily induced that environmental complexity has a positive impact on enterprises’ green innovation activities, while environmental dynamism has a negative impact on enterprises’ green innovation activities. A further VIF test is carried out on the main variables. It can be seen from Table 4 that the VIF values of the variables are all less than 10, indicating that there is no multicollinearity problem among the variables.

### 4.3. Multiple Regression Analysis

#### 4.3.1. Regression Analysis of Environmental Uncertainty and Enterprises’ Green Technological Innovation

Table 5 shows the regression results of environmental uncertainty and enterprises’ green technological innovation. As can be seen from column (1), the regression coefficient between environmental uncertainty and enterprises’ green technological innovation (ln*GI*) is 0.0836, which is significantly positive at the level of 1%, indicating that environmental uncertainty stimulates enterprises to carry out green technological innovation, H1a is verified. In column (2) of Table 5, the regression coefficient environmental complexity (*HHI*) and enterprises’ green technological innovation (ln*GI*) is 0.125, which is significantly positive, indicating that when the market competition is more intense, the enterprises will strengthen green technological innovation to gain more market profits, H1b is verified. As can be seen from column (3), environmental dynamism (*EU_adj*) and enterprises’ green technological innovation (ln*GI*) are significantly negatively correlated at a 1% confidence level, with a regression coefficient of −0.0102, indicating that environmental dynamism has a restraining effect on enterprises’ green technological innovation. In other words, enterprises often adopt a negative attitude towards green technological innovation activities and choose to postpone or give up innovation investment when facing obvious fluctuations in sales revenue; H1c is verified.

Due to China’s special national conditions, the policies’ strength on state-owned enterprises (SOEs) and non-state-owned enterprises (Non-SOEs) are different, and the enterprises’ responses are also different. Therefore, we check whether there are differences in the relationship between environmental uncertainty and green technological innovation in SOEs and Non-SOEs.

Table 6 and Table 7 show the regression results of the environmental uncertainty and the green technological innovation in SOEs and Non-SOEs, respectively. As can be seen from column (1) of Table 6 and Table 7, in SOEs, environmental uncertainty and enterprises’ green technological innovation are significantly positive at the level of 1%, and environmental uncertainty and enterprises’ green technological innovation in non-SOEs are significantly positive at the level of 10%. In general, the motivation of environmental uncertainty for enterprises’ green technological innovation is more obvious in SOEs. As can be seen from column (2) of Table 6 and Table 7, environmental complexity and enterprises’ green technological innovation in SOEs is significantly positive at the level of 5%, and the regression coefficient between environmental complexity and enterprises’ green technological innovation in Non-SOEs is smaller than that in SOEs, which is not significant, indicating that SOEs will be more active in green technological innovation than Non-SOEs in the external environment of fierce market competition. It can be seen from column (3) of Table 6 and Table 7 that environmental dynamism and enterprises’ green technological innovation in SOE is significantly negative at the level of 5%, while environmental dynamism and enterprises’ green technological innovation in non-SOEs is not significant. It shows that compared with non-SOEs, when SOEs are faced with fluctuations in sales revenue, they are more likely to reduce their green innovation.

#### 4.3.2. Regression Analysis of Environmental Uncertainty, Environmental Regulation and Enterprises’ Green Technological Innovation

Table 8 shows the moderating effect of environmental regulation on environmental uncertainty and enterprises’ green technological innovation. As can be seen from column (1) in Table 8, at the level of 1%, environmental regulation (*ER*) is significantly positively correlated with enterprises’ green technological innovation (ln*GI*), with a regression coefficient of 0.0518, manifesting the motivation of environmental regulation for enterprises’ green technological innovation. H2 is verified. Column (2) of Table 8 adds the interaction term between environmental regulation (*ER*) and environmental uncertainty (*EU*). It can be seen that the environmental uncertainty and enterprises’ green technological innovation are significantly positive at 1% level, with a regression coefficient of 0.176. The interaction term *EU* × *ER* between environmental uncertainty and environmental regulation is significantly positive at the 1% level, with a regression coefficient of 0.126. This indicates that the environmental uncertainty can enhance the promotion of enterprises’ green technological innovation through high environmental regulation. H3a is verified. The interaction item between environmental regulation and environmental complexity is added to column (3) in Table 8, and *HHI* × *ER* is significantly positive at a confidence level of 10%, with a regression coefficient of 0.128, indicating that the greater the environmental complexity faced by the enterprise, the more obvious the incentive effect on the enterprises’ green technological innovation will be, and indirectly strengthening the promotion effect on the enterprises’ green technological innovation through environmental regulation. H3b is verified. As can be seen from column (3) in Table 8, *EU_adj* × *ER* has a significant negative correlation at 1% level after the interaction between environmental regulation and environmental dynamism is added, with a regression coefficient of −0.0313. This indicates that if the environmental policy monitoring efforts faced by enterprises are strengthened, the inhibition of environmental dynamism on enterprises’ green technological innovation activities will be deepened, thus reducing the patent output of enterprises and weakening their competitiveness. H3c is verified.

Based on the heterogeneity of property rights, we further analyze the relationship between environmental uncertainty, environmental regulation and enterprises’ green technological innovation.

Table 9 and Table 10 verify whether the moderating effect of environmental regulation on environmental uncertainty and enterprises’ green technological innovation is different based on different property rights. As can be seen from column (1) of Table 9 and Table 10, the regression coefficient between the environmental regulation of SOEs and enterprises’ green technological innovation is 0.0796, which is significantly positive at the level of 1%, while that of non-SOEs is significantly positive at the level of 5%, with a regression coefficient of 0.0313, which is lower than the correlation coefficient of SOEs, indicating that SOEs subject to environmental regulation are more conducive to promoting green technological innovation; that is, SOEs are more responsive to the environmental regulation of the government, which will increase the improvement of green technological innovation of their own enterprises and strengthen the hard power of enterprises. The reason may be that SOEs, backed by the state and the government, enjoy greater preferential policies and therefore should take more social responsibilities. Column (2) in Table 9 and Table 10 shows the moderating effect of environmental regulation on environmental uncertainty and green technological innovation of state-owned and non-SOEs. It can be seen that both state-owned and non-SOEs, *EU* × *ER,* are significantly positive at 1%, but the correlation coefficient of SOEs is greater than that of non-SOEs, indicating that environmental regulation has a stronger incremental effect on environmental uncertainty of SOEs and enterprises’ green technological innovation. The regression coefficient of state-owned enterprise *HHI* × *ER* is 0.0149, but is not significantly positive, indicating that compared with non-SOEs, environmental regulation will strengthen the promotion of green technological innovation for SOEs facing higher environmental complexity. This is because when the industry competition is more intense, that is, the industry access threshold is lower, the SOEs, with their unique political advantages, abundant sources of funds and larger enterprises’ scale, are less impacted by the new enterprises and have more energy and funds to invest in green technological innovation. However, non-SOEs have limited funds and long-term financing difficulties. Facing the increasingly diversified green consumption demand of consumers, they are unable to carry out more green technological innovation. *EU_adj* × *ER* of Non-SOEs is significantly negative at the level of 1%, with a regression coefficient of −0.0481, but it is not significant among the SOEs, indicating that compared with the SOEs, non-SOEs with large fluctuations in sales revenue will reduce enterprises’ innovation in the face of environmental regulations. Non-SOEs usually face the dilemma of financing difficulties due to their property rights, and the increase in the intensity of environmental regulations increases the daily cost of environmental protection for enterprises, increases the operating costs of enterprises and makes innovation activities, which are due to lack of financial support, difficult.

#### 4.3.3. The Lag Effect Test of Environmental Uncertainty, Environmental Regulation and Enterprises’ Green Technological Innovation

##### The Lag Effect Test of Environmental Uncertainty on Enterprises’ Green Technological Innovation

It takes time for environmental uncertainty and environmental regulation to take effect. The number of green patent applications filed by enterprises in the current period may be affected by the uncertainties in previous years and the environmental policies promulgated. Therefore, in order to test whether the relationship among environmental uncertainty, environmental regulation and enterprises’ green technological innovation have a lag effect, this paper tests the lag effect of the relationship among the three by lagging enterprises’ green technological innovation by one stage and two stages, respectively, to reduce the impact of endogeneity on the empirical results. As can be seen from column (1) in Table 11, environmental uncertainty (*EU*) is significantly positive at a confidence level of 1%, which indicates that the promotion of environmental uncertainty on enterprises’ green technological innovation can last for one year.

Column (1) of Table 11 shows that environmental uncertainty (*EU*) is significantly positive at a confidence level of 1%, which indicates that the promotion of environmental uncertainty on enterprises’ green technological innovation can last for one year. As can be seen from column (2) in Table 11, at a confidence level of 10%, the environmental complexity (*HHI*) is significantly positive with the enterprises’ green technological innovation lagging behind by one stage (L.ln*GI*), with a regression coefficient of 0.0917, indicating that the environmental complexity can promote enterprises’ green technological innovation, and the effect can last for one year. From column (3) in Table 11, it can be seen that environmental dynamism (*EU_adj*) is significantly negative to enterprises’ green technological innovation lagging behind by one stage (L.ln*GI*) at the level of 10%, with a regression coefficient of −0.00721, indicating that environmental dynamism has inhibitory effect on enterprises’ green technological innovation, and this effect can last for one year.

Table 12 examines the relationship among the overall effect of environmental uncertainty, environmental complexity and environmental dynamism, and the two-stage lag in enterprises’ green technological innovation. As can be seen from column (1) in Table 12, the regression coefficient between environmental uncertainty (*EU*) and enterprises’ green technological innovation lagging behind two periods (L2.ln*GI*) is 0.0771, which is significantly positive at the confidence level of 1%, indicating that the promotion effect of environmental uncertainty on enterprises’ green technological innovation can last for two years. As can be seen from column (2) in Table 12, at a confidence level of 10%, the environmental complexity (*HHI*) is significantly positive with the enterprises’ green technological innovation lagging behind by two periods (L2.ln*GI*), with a regression coefficient of 0.0866, indicating that the promotion effect of environmental complexity on the enterprises’ green technological innovation can be maintained for two years. From column (3) in Table 12, it can be seen that the environmental dynamism (*EU_adj*) and the enterprises’ green technological innovation lagging behind two periods (L2.ln*GI*) are significantly negative at the level of 10%, with a regression coefficient of −0.00721, indicating that the inhibition effect of environmental dynamism on enterprises’ green technological innovation can last for two years.

##### The Lag Effect of Environmental Uncertainty, Environmental Regulation and Enterprises’ Green Technological Innovation

Table 13 shows the results of the lag effect of environmental uncertainty, environmental regulation, and enterprises’ green technological innovation under the condition of lagging green technological innovation by one stage. As can be seen from column (1), environmental regulation (*ER*) and enterprises’ green technological innovation lagging behind by one stage (L.ln*GI*) are also significantly positive at the level of 1%, indicating that environmental regulation can promote enterprises’ green technological innovation and the effect can last for one year. Column (2) in Table 13 introduces the interaction term between environmental regulation and environmental uncertainty. It can be seen that *EU* × *ER* is significantly positive at the level of 1%, and the regression coefficient is 0.144, indicating that the incremental effect of environmental regulation on environmental uncertainty and enterprises’ green technological innovation can last for one year. In column (3) of Table 13, the interaction term *HHI* × *ER* between environmental complexity and environmental regulation is added, which is significantly positive at a confidence level of 1%, indicating that the promotion effect of environmental regulation on enterprises’ green technological innovation through environmental complexity can last for one year. The interaction term *EU_adj* × *ER* between environmental dynamism and enterprises’ green technological innovation is added to column (4) of Table 13, with a regression coefficient of −0.0369, which is significantly negative at 1%, indicating that environmental regulation can intensify the inhibitory effect on enterprises’ green technological innovation through environmental dynamism for one year.

Table 14 shows the results of the lag effect of environmental uncertainty, environmental regulation, and enterprises’ green technological innovation under the lag of two periods. From column (1), environmental regulation and enterprises’ green technological innovation are significantly positive at 1%, indicating that the promotion effect of environmental regulation on enterprises’ green technological innovation can last for two years. It can be seen from column (2) that *EU* × *ER* is significantly positive, and the environmental uncertainty is positive at the level of 1% for enterprises’ green technological innovation that lag behind two periods; the results are consistent with the previous, indicating that the positive moderating effect of environmental regulation on environmental uncertainty and enterprises’ green technological innovation is still effective after lagging behind two periods of enterprises’ green technological innovation. In Table 14, columns (3) and (4) for the test of the moderating effect of environmental regulation on environmental complexity, environmental dynamism and enterprises’ green technological innovation are lagging by two periods, respectively. The regression results are consistent with the previous ones and will not be described in more detail. Based on the above analysis results of lag effect, it can be concluded that the moderating effect of environmental regulation on environmental uncertainty and enterprises’ green technological innovation, whether strengthened or intensified, will last for two years.

### 4.4. Robustness Analysis

Based on the methods of robustness analysis in the existing literature, this paper chooses the method of replacing variables and replacing measurement models to conduct in-depth research to test whether the regression results in the previous section will change accordingly, and then to confirm the reliability of the research results in this paper.

#### 4.4.1. Variables Substitution

##### Replace Enterprises’ Green Technological Innovation Variables

This paper replaces the enterprises’ green technological innovation variable (ln*GI*) with the logarithm of the sum of the authorizations of green invention patents and green utility model patents (ln*GI*1), because the patent authorizations are the final green technological innovation results obtained by the enterprise. Taking this as an alternative variable, the relationship between environmental uncertainty, environmental regulation and enterprises’ green technological innovation can be analyzed more intuitively, and the credibility of the regression results can be double confirmed. Table A1 in Appendix A shows the robustness test of environmental uncertainty and enterprises’ green technological innovation. Compared with the multivariate regression results in Table 5, the sign in front of the key variable coefficient is consistent with the hypothesis, and the difference in P value is small, indicating that the regression analysis of environmental uncertainty and enterprises’ green technological innovation is robust. Table A2 in Appendix A presents the robustness test result of environmental regulation, environmental uncertainty and enterprises’ green technological innovation. By comparing with the regression results in Table 6, it is consistent with the previous conclusions and enhances the robustness of the regression results in this paper.

##### Replace Environmental Uncertainty Variables

In order to ensure the consistency of the results and the robustness of the model, the Herfindal–Hirschman Index (*HHI*1), which takes operating income as the indicator in the negative processing place, is used as the substituting indicator of the complexity of environmental uncertainty instead of the Herfindal–Hirschman Index, which takes main operating income as the indicator in the negative processing place, and the fluctuation of sales income (*EU*_unadj), which is not adjusted by the industry, is used as the replacement indicator of the dynamism of the environmental uncertainty. As can be seen from Table A3 in Appendix A, the robustness test result of environmental uncertainty and enterprises’ green technological innovation is consistent with the previous results. Compared with Table 6, it can be seen from Table A4 in Appendix A that the sign before the coefficient is consistent, and the change of *p* value is small, which indicates that the above analysis of the moderating effect of environmental regulation is more reliable.

The dependent able variable enterprises’ green technological innovation is directly measured by the number of enterprises’ green patent applications, which is expressed as *GI*. There are many zero values in the number of enterprises’ green patent applications. According to this data characteristic, it is more appropriate to use the Poisson model to test, which also increases the credibility of the research results.

#### 4.4.2. Replace the Measurement Model

As can be seen from Table A5 in Appendix A, in the Poisson model, the environmental uncertainty and environmental complexity have a positive correlation with the number of patent applications (*GI*), which is significant at the level of 1%, while the environmental dynamism have a significant negative correlation with the number of patent applications (*GI*), which is consistent with the previous results. According to column (1) in Table A6 in Appendix A, the environmental regulation and the number of patent applications are significantly positive at a confidence level of 1%, indicating that the more stringent the environmental regulation, the better the promotion of enterprises’ green innovation and the increase in the number of patent applications. Column (2) of Table A6 shows that the interaction term *EU* × *ER* between environmental regulation and environmental uncertainty is significantly positive, which is consistent with the previous results. Column (3) of Table A6 represents the interaction term *HHI* × *ER* between environmental regulation and environmental complexity is significantly positive, which is consistent with the previous results. Column (4) of Table A6 manifests the interaction term *EU_adj* × *ER* between environmental regulation and environmental dynamism is significantly negative, which is consistent with the previous results. In a word, even if environmental regulation will intensify the inhibition of environmental dynamism on enterprises’ green technological innovation, environmental complexity reverses the inhibition, which results in the overall environmental regulation can produce incremental effects on environmental uncertainty and enterprises’ green technological innovation. This conclusion is consistent with the previous conclusion, strengthening the stability of the research conclusion in this paper.

## 5. Conclusions, Policy Implications, Limitations and Future Prospects

### 5.1. Conclusions

Based on the empirical data of Shanghai and Shenzhen A-share listed companies from 2005 to 2019, this paper employs two dimensions, environmental complexity and environmental dynamism, rather than the traditional single environmental dynamism to measure environmental uncertainty to explore the relationship among environmental uncertainty, environmental regulation, and enterprises’ green technological innovation. After in-depth analysis, it is concluded that, first, overall, the environmental uncertainty formed under the joint action of environmental complexity and dynamism has a promoting effect on enterprises’ green technological innovation; that is, the greater the environmental uncertainty, the more it can promote enterprises’ green technological innovation. For environmental complexity, the fiercer the market competition the enterprise faces, the more it can promote the enterprises’ green technological innovation. However, when environmental dynamism increases, it will hinder enterprises’ green technological innovation activities. Further analysis shows that the effect of environmental uncertainty on green technological innovation will last for 2 years. This finding complements the research on the effect of environmental uncertainty, which is measured in two dimensions on enterprises’ green technological innovation.

Second, the “compensation effect” of environmental regulation and enterprises’ green technological innovation offsets the “crowding-out effect”. Enterprises produce products that meet the public’s green demand through green technological innovation and reduce production costs at the same time, indicating that environmental regulation can significantly promote enterprises’ green technological innovation. Further discussion shows that the promotion effect of environmental regulation on enterprises’ green technological innovation will last for 2 years. This finding also corroborates the study by Zhao and Sun (2016).

Third, environmental regulation has a moderating effect on environmental uncertainty and enterprises’ green technological innovation. With the two dimensions environmental complexity and dynamism, the interaction between environmental regulation and environmental uncertainty is added to the regression model. The empirical analysis shows that, on the whole, environmental regulation accentuates the impact of environmental uncertainty on enterprises’ green technological innovation; that is, environmental uncertainty accelerates enterprises’ green technological innovation through strict environmental regulation. Specifically, environmental regulation can further strengthen the promotion effect of environmental complexity on enterprises’ green technological innovation. Environmental dynamism deepens the inhibition of enterprises’ green technological innovation through environmental regulation. Further research shows that the moderating effect of environmental regulation on environmental uncertainty and enterprises’ green technological innovation can last for 2 years.

Fourthly, based on the heterogeneity of property rights, the relationship among environmental uncertainty, environmental regulation and enterprises’ green technological innovation is analyzed in depth, and a conclusion is drawn: compared with non-SOEs, environmental uncertainty and environmental regulation in SOEs play a more prominent role in promoting enterprises’ green technological innovation. When SOEs face increased environmental uncertainty, fierce market competition and strict environmental regulation, the more it can stimulate enterprises’ green technological innovation activities, and at the same time, environmental dynamism plays a more significant role in inhibiting green technological innovation. Further analysis shows that the positive moderating effect of environmental regulation on environmental uncertainty and enterprises’ green technological innovation is more significant in SOEs, and the negative moderating effect on environmental dynamism and enterprises’ green technological innovation is more significant in non-SOEs.

### 5.2. Policy Implications

From the perspective of environmental complexity and dynamism, this paper analyzes the status of environmental uncertainty, environmental regulation, and green technological innovation in China, and empirically tests the relationship among them. It can be concluded that all the environmental uncertainty, environmental complexity, and environmental regulation have significant promotion effect on enterprises’ green technological innovation, and environmental dynamism has inhibitory effect on enterprises’ green technological innovation. It is further found that environmental regulation has a moderating effect on the relationship between environmental uncertainty and enterprises’ green technological innovation. Based on the research conclusions and the specific national conditions of our country, the following suggestions are proposed.

First, rational, and detailed environmental regulation standards need to be established and support for enterprises’ innovation needs to be increased. Based on the heterogeneity factors such as the fluctuation of external market environment and the degree of competition, a differentiated management strategy should be implemented, appropriate punishment measures should be taken, and both rewards and punishments should be implemented to give full play to the advantages of national policy as a tangible influence. Environmental regulation can significantly stimulate enterprises’ green technological innovation, and environmental uncertainty will have a moderating effect on the relationship between the two. Therefore, the government should adopt incentive policies, such as subsidies, to provide funds for energy conservation and emission reduction of enterprises, to ease the financing difficulties of enterprises, especially small and medium-sized enterprises, to improve efficiency, and to promote enterprises’ green technological innovation when enterprises are facing large fluctuations in environmental dynamism. In addition, due to the different property rights of enterprises, environmental policies should be made on a “person-by-person” basis, avoiding a “one size fits all” policy, strengthening the control and support of non-SOEs, and maintaining supervision over SOEs.

Second, a fair, open, and transparent industry atmosphere should be created, and the market’s leadership should be strengthened. The complexity of the intensive external environment, such as the high degree of competition in the industry, may not totally bad for enterprises, it can sometimes motivate enterprises to carry out green technological innovation. Based on the incremental effect of environmental complexity on environmental regulation and enterprises’ green technological innovation, we should link the tangible influence of the government and its appropriate environmental regulation with the intangible influence of the market, guide the establishment of a market innovation environment with good quality through appropriate environmental policies, point out the direction for various market participants and promote enterprises’ green technological innovation.

Third, a sound internal enterprises’ governance mechanism and information disclosure mechanism should be set up. Enterprises need to face increasing uncertainty and fluctuations of the external market environment, and such fluctuations are unpredictable and random, which requires enterprises to improve the handling efficiency and control ability of uncertainty, comply with the environmental policies proposed by the state, guide enterprises to develop in a positive and innovation-driven direction, and promote enterprises’ green technological innovation, with the goal of maximizing enterprises’ value. At the same time, the information of the enterprise should be transparent enough to provide useful and timely information to external investors, thus improving the efficiency of external financing, alleviating the problem of information asymmetry and providing financial support for the enterprises’ green technological innovation.

### 5.3. Limitations and Future Prospects

This paper has some contributions on enriching the research on the economic outcome of environmental uncertainty and environmental regulation. However, this paper still has shortcomings: on one hand, the measurement of environmental uncertainty needs to be further deepened to more dimensions. On the other hand, environmental regulation can be measured by different types of environmental policies, and the impact of different environmental policies on enterprises’ green technological innovation and the mechanism of the relationship between environmental uncertainty and enterprises’ green technological innovation should be discussed.

## Figures and Tables

**Table 1:** Definition of Main Variables.

Type	Name	Symbol	Definition
Explained Variable	Green Technological Innovation	ln*GI*	The natural logarithm of the sum of green invention patents and green utility model patents.
Explanatory Variable	Environmental Uncertainty	*EU*	Interaction between complexity of environment and dynamics of environment.
	Environmental Complexity	*HHI*	Herfindahl–Hirschman index with the main business income as the index, and negative processing.
	Environmental Dynamism	*EU_adj*	Industry-adjusted sales revenue fluctuations.
Moderator	Environmental Regulation	*ER*	The intensity of environmental regulation calculated by comprehensive index method.
Control Variable	Scale of Corporation	Size	The natural logarithm of total assets of corporation.
	Corporate social value	ln*TobinQ*	The ratio of the sum of the market value of owners’ equity and liabilities to the total book assets.
	Asset–liability ratio	*Lev*	The ratio of total liabilities to total assets in the balance sheet disclosed at the end of the year.
	The largest shareholder’s shareholding ratio	Largest	The ratio of the number of shares held by the largest shareholder to the total number of shares.
	Executive shareholding ratio	*CGB*	The ratio of the number of executives holding shares to the total share capital.
	Proportion of independent directors	Dud	The ratio of the number of independent directors to the total number of board members.
	Return on Total Assets	*ROA*	Proportion of net profit of corporations to average total assets.
	Dual Role of the Board Chairman	Dual	Whether the general manager is also the chairman.
	Industry	Industry	Industry is an industry dummy variable.
	Year	Year	Year is the annual dummy variable.

**Table 2:** Descriptive statistics of each variable.

Variable	Obs	Mean	Std. Dev.	Median	Min	Max
ln*GI*	21,359	0.398	0.831	0	0	3.850
*EU*	21,359	−0.171	0.238	−0.088	−1.470	−0.007
*HHI*	21,359	−0.134	0.134	−0.090	−0.793	−0.020
*EU_adj*	21,359	1.287	1.144	0.962	0.130	6.705
*ER*	21,359	0.693	0.608	0.591	0	2.179
Size	21,359	22.29	1.259	22.14	19.83	26.11
*TobinQ*	21,359	1.977	1.242	1.559	0.875	7.998
*ROA*	21,359	0.032	0.060	0.031	−0.253	0.193
*Lev*	21,359	0.475	0.199	0.481	0.069	0.902
Largest	21,359	35.18	15.00	33.13	9.229	74.82
Dual	21,359	0.195	0.396	0	0	1
*CGB*	21,359	0.042	0.100	0	0	0.446
Dud	21,359	37.01	5.251	33.33	28.57	57.14

Notes: a. Obs. Denotes the number of observations. b. Std. Dev. Indicates standard deviation.

**Table 3:** Correlation analysis of the main variables.

	ln*GI*	*EU*	*HHI*	*EU_adj*	*ER*	Size	*TobinQ*	*ROA*	*Lev*	Largest	Dual	*CGB*	Dud
ln*GI*	1												
*EU*	0.07 ***	1											
*HHI*	0.02 ***	0.66 ***	1										
*EU_adj*	−0.08 ***	−0.61 ***	−0.01 *	1									
*ER*	0.06 ***	0.05 ***	0.05 ***	−0.03 ***	1								
Size	0.26 ***	−0.01	−0.06 ***	−0.06 ***	−0.03 ***	1							
*TobinQ*	−0.05 ***	−0.03 ***	0.01	0.05 ***	−0.04 ***	−0.43 ***	1						
*ROA*	0.04 ***	0.06 ***	0.01	−0.08 ***	0.01 *	0.08 ***	0.15 ***	1					
*Lev*	0.05 ***	−0.03 ***	−0.01	0.04 ***	−0.02 ***	0.39 ***	−0.33 ***	−0.34 ***	1				
Largest	0.01	−0.04 ***	−0.06 ***	0.01 *	−0.06 ***	0.24 ***	−0.12 ***	0.12 ***	0.09 ***	1			
Dual	0.02 ***	0.01	0.03 ***	0.01 *	0.03 ***	−0.10 ***	0.10 ***	−0.01	−0.09 ***	−0.10 ***	1		
*CGB*	0.08 ***	0.02 ***	0.04 ***	−0.01	0.07 ***	−0.15 ***	0.09 ***	0.05 ***	−0.25 ***	−0.16 ***	0.21 ***	1	
Dud	0.03 ***	−0.01	−0.00	0.01 **	−0.03 ***	0.05 ***	0.05 ***	−0.03 ***	−0.01 **	0.01 *	0.11 ***	0.08 ***	1

*** *p* < 0.01, ** *p* < 0.05, * *p* < 0.1.

**Table 4:** VIF test of main variables.

Variable	VIF
*EU*	4.96
*ER*	1.02
*HHI*	3.13
*EU_adj*	2.81
Size	1.53
*TobinQ*	1.32
*ROA*	1.26
*Lev*	1.50
Large	1.11
Dual	1.07
*CGB*	1.14
Dud	1.03
Mean of VIF	1.82

**Table 5:** Regression analysis of environmental uncertainty and enterprises’ green technological innovation.

Variable	(1)	(2)	(3)
	ln*GI*	ln*GI*	ln*GI*
*EU*	0.0836 ***		
	(0.0198)		
*HHI*		0.125 ***	
		(0.0436)	
*EU_adj*		A	−0.0102 ***
			(0.00374)
Size	0.0530 ***	0.0508 ***	0.0514 ***
	(0.00900)	(0.00898)	(0.00899)
*TobinQ*	−0.00825 *	−0.00838 *	−0.00835 *
	(0.00478)	(0.00478)	(0.00478)
*ROA*	−0.0148	−0.0195	−0.0130
	(0.0779)	(0.0779)	(0.0780)
*Lev*	0.0607 *	0.0569	0.0636 *
	(0.0367)	(0.0367)	(0.0367)
Largest	−0.00357 ***	−0.00368 ***	−0.00359 ***
	(0.000539)	(0.000538)	(0.000540)
Dual	−0.0246 *	−0.0241 *	−0.0249 *
	(0.0129)	(0.0129)	(0.0129)
*CGB*	0.0566	0.0667	0.0619
	(0.104)	(0.104)	(0.105)
Dud	0.00138	0.00143	0.00135
	(0.000987)	(0.000988)	(0.000988)
Constant	−0.847 ***	−0.794 ***	−0.813 ***
	(0.192)	(0.191)	(0.192)
Observation	21,359	21,359	21,359
Adjusted R^2^	0.059	0.058	0.058
Industry, Year	Control		

*t*-statistics in parentheses. *** *p* < 0.01 * *p* < 0.1.

**Table 6:** Regression Analysis of Environmental Uncertainty and Green Technological Innovation of SOEs.

Variable	(1)	(2)	(3)
	ln*GI*	ln*GI*	ln*GI*
*EU*	0.0897 ***		
	(0.0273)		
*HHI*		0.118 **	
		(0.0574)	
*EU_adj*			−0.0129 **
			(0.00516)
Size	0.0443 ***	0.0410 ***	0.0440 ***
	(0.0128)	(0.0127)	(0.0128)
*TobinQ*	−0.0160 **	−0.0163 **	−0.0157 **
	(0.00730)	(0.00731)	(0.00731)
*ROA*	−0.0679	−0.0731	−0.0625
	(0.121)	(0.121)	(0.121)
*Lev*	0.104 **	0.0991 *	0.110 **
	(0.0519)	(0.0520)	(0.0519)
Largest	−0.00441 ***	−0.00453 ***	−0.00441 ***
	(0.000743)	(0.000742)	(0.000744)
Dual	−0.0352 *	−0.0331	−0.0364 *
	(0.0204)	(0.0204)	(0.0204)
*CGB*	0.349	0.347	0.387
	(0.826)	(0.826)	(0.826)
Dud	0.00272 **	0.00274 **	0.00269 **
	(0.00126)	(0.00126)	(0.00126)
Constant	−0.710 ***	−0.629 **	−0.705 ***
	(0.273)	(0.272)	(0.273)
Observation	11,238	11,238	11,238
Adjusted R^2^	0.086	0.085	0.085
Industry, Year	Control		

*t*-statistics in parentheses. *** *p* < 0.01, ** *p* < 0.05, * *p* < 0.1.

**Table 7:** Regression Analysis of Environmental Uncertainty and Green Technological Innovation of Non-SOEs.

Variable	(1)	(2)	(3)
	ln*GI*	ln*GI*	ln*GI*
*EU*	0.0509 *		
	(0.0303)		
*HHI*		0.0991	
		(0.0701)	
*EU_adj*			−0.00492
			(0.00566)
Size	0.0720 ***	0.0720 ***	0.0706 ***
	(0.0141)	(0.0141)	(0.0141)
*TobinQ*	0.00348	0.00380	0.00326
	(0.00668)	(0.00668)	(0.00668)
*ROA*	0.0266	0.0222	0.0268
	(0.107)	(0.107)	(0.107)
*Lev*	−0.0200	−0.0232	−0.0191
	(0.0553)	(0.0553)	(0.0553)
Largest	−0.00211 **	−0.00217 **	−0.00214 **
	(0.000865)	(0.000864)	(0.000867)
Dual	−0.0231	−0.0235	−0.0231
	(0.0175)	(0.0175)	(0.0175)
*CGB*	0.0158	0.0232	0.0192
	(0.111)	(0.111)	(0.111)
Dud	−0.000231	−0.000146	−0.000254
	(0.00166)	(0.00166)	(0.00166)
Constant	−1.164 ***	−1.158 ***	−1.133 ***
	(0.297)	(0.297)	(0.296)
Observation	10,121	10,121	10,121
Adjusted R^2^	0.034	0.034	0.034
Industry, Year	Control		

*t*-statistics in parentheses. *** *p* < 0.01, ** *p* < 0.05, * *p* < 0.1.

**Table 8:** Regression Analysis of Environmental Uncertainty, Environmental Regulation and Enterprises’ Green Technological Innovation.

Variable	(1)	(2)	(3)	(4)
	ln*GI*	ln*GI*	ln*GI*	ln*GI*
*ER*	0.0518 ***	0.0492 ***	0.0591 ***	0.0892 ***
	(0.00902)	(0.00897)	(0.0198)	(0.0132)
*EU*		0.176 ***		
		(0.0212)		
*EU* × *ER*		0.126 ***		
		(0.0304)		
*HHI*			0.146 ***	
			(0.0451)	
*HHI* × *ER*			0.128 *	
			(0.0725)	
*EU_adj*				−0.0178 ***
				(0.00547)
*EU_adj* × *ER*				−0.0313 ***
				(0.00699)
Size	0.202 ***	0.199 ***	0.0507 ***	0.199 ***
	(0.00726)	(0.00727)	(0.00898)	(0.00726)
*TobinQ*	0.0270 ***	0.0278 ***	−0.00814 *	0.0284 ***
	(0.00481)	(0.00481)	(0.00478)	(0.00481)
*ROA*	0.443 ***	0.422 ***	−0.0228	0.394 ***
	(0.0902)	(0.0902)	(0.0779)	(0.0898)
*Lev*	−0.00223	0.0105	0.0530	0.0151
	(0.0319)	(0.0319)	(0.0367)	(0.0318)
Largest	−0.000966 ***	−0.000925 **	−0.00362 ***	−0.000858 **
	(0.000367)	(0.000367)	(0.000538)	(0.000366)
Dual	0.00835	0.00828	−0.0234 *	0.00766
	(0.0133)	(0.0133)	(0.0129)	(0.0133)
*CGB*	0.140 **	0.142 **	0.0731	0.135 **
	(0.0612)	(0.0612)	(0.104)	(0.0611)
Dud	0.000472	0.000412	0.00147	0.000534
	(0.00102)	(0.00102)	(0.000987)	(0.00102)
Constant	−4.421 ***	−4.337 ***	−0.834 ***	−4.360 ***
	(0.158)	(0.159)	(0.192)	(0.159)
Observation	21,359	21,359	21,359	21,359
Adjusted R^2^	0.246	0.248	0.059	0.249
Industry, Year	Control			

*t*-statistics in parentheses. *** *p* < 0.01, ** *p* < 0.05, * *p* < 0.1.

**Table 9:** Regression Analysis of Environmental Uncertainty, Environmental Regulation and Enterprises’ Green Technological Innovation of SOEs.

Variable	(1)	(2)	(3)	(4)
	ln*GI*	ln*GI*	ln*GI*	ln*GI*
*ER*	0.0796 ***	0.0752 ***	0.0955 ***	0.0769 ***
	(0.0128)	(0.0128)	(0.0139)	(0.0116)
*EU*		0.143 ***		
		(0.0307)		
*EU* × *ER*		0.164 ***		
		(0.0507)		
*HHI*			0.312 ***	
			(0.0517)	
*HHI* × *ER*			0.746 ***	
			(0.104)	
*EU_adj*				−0.0387 ***
				(0.00637)
*EU_adj* × *ER*				−0.0153
				(0.0108)
Size	0.207 ***	0.205 ***	0.211 ***	0.204 ***
	(0.00930)	(0.00934)	(0.0102)	(0.00740)
*TobinQ*	0.0151 **	0.0162 **	0.0530 ***	0.0174 **
	(0.00750)	(0.00749)	(0.00798)	(0.00811)
*ROA*	0.127	0.104	−0.539 ***	0.0841
	(0.145)	(0.146)	(0.150)	(0.149)
*Lev*	−0.234 ***	−0.222 ***	−0.217 ***	−0.217 ***
	(0.0453)	(0.0454)	(0.0459)	(0.0461)
Largest	−0.00226 ***	−0.00222 ***	−0.00146 ***	−0.00208 ***
	(0.000496)	(0.000495)	(0.000548)	(0.000491)
Dual	0.0377 *	0.0377 *	0.000359	0.0338
	(0.0217)	(0.0217)	(0.0242)	(0.0229)
*CGB*	0.0435	0.0650	1.989 ***	0.0879
	(0.572)	(0.571)	(0.601)	(0.449)
Dud	0.00189	0.00188	0.00123	0.00194
	(0.00143)	(0.00143)	(0.00158)	(0.00133)
Constant	−4.510 ***	−4.445 ***	−4.405 ***	−4.434 ***
	(0.200)	(0.201)	(0.216)	(0.173)
Observation	11,238	11,238	11,238	11,238
Adjusted R^2^	0.298	0.299	0.120	0.300
Industry, Year	Control			

*t*-statistics in parentheses. *** *p* < 0.01, ** *p* < 0.05, * *p* < 0.1.

**Table 10:** Regression Analysis of Environmental Uncertainty, Environmental Regulation and Enterprises’ Green Technological Innovation of Non-SOEs.

Variable	(1)	(2)	(3)	(4)
	ln*GI*	ln*GI*	ln*GI*	ln*GI*
*ER*	0.0313 **	0.0277 **	0.0663 ***	0.0304 **
	(0.0131)	(0.0130)	(0.0134)	(0.0127)
*EU*		0.203 ***		
		(0.0303)		
*EU* × *ER*		0.136 ***		
		(0.0395)		
*HHI*			0.255 ***	
			(0.0560)	
*HHI* × *ER*			0.0149	
			(0.0923)	
*EU_adj*				−0.0387 ***
				(0.00628)
*EU_adj* × *ER*				−0.0481 ***
				(0.0104)
Size	0.199 ***	0.197 ***	0.167 ***	0.195 ***
	(0.0119)	(0.0119)	(0.0124)	(0.00981)
*TobinQ*	0.0381 ***	0.0385 ***	0.0341 ***	0.0380 ***
	(0.00655)	(0.00653)	(0.00663)	(0.00719)
*ROA*	0.651 ***	0.628 ***	0.573 ***	0.598 ***
	(0.117)	(0.116)	(0.120)	(0.129)
*Lev*	0.179 ***	0.190 ***	0.154 ***	0.203 ***
	(0.0452)	(0.0452)	(0.0450)	(0.0499)
Largest	−0.000336	−0.000258	−0.00175 ***	−0.000249
	(0.000589)	(0.000588)	(0.000616)	(0.000570)
Dual	0.0231	0.0223	0.0481 ***	0.0208
	(0.0170)	(0.0170)	(0.0180)	(0.0164)
*CGB*	0.379 ***	0.369 ***	0.712 ***	0.356 ***
	(0.0674)	(0.0674)	(0.0685)	(0.0639)
Dud	−0.000242	−0.000375	−0.00168	−0.000334
	(0.00144)	(0.00144)	(0.00153)	(0.00143)
Constant	−4.229 ***	−4.135 ***	−3.464 ***	−4.128 ***
	(0.265)	(0.265)	(0.271)	(0.239)
Observation	10,121	10,121	10,121	10,121
Adjusted R^2^	0.229	0.231	0.079	0.233
Industry, Year	Control			

*t*-statistics in parentheses. *** *p* < 0.01, ** *p* < 0.05.

**Table 11:** Regression Analysis of Environmental Uncertainty and Enterprises’ Green Technological Innovation Lagging One Period.

Variable	(1)	(2)	(3)
	L.ln*GI*	L.ln*GI*	L.ln*GI*
*EU*	0.0766 ***		
	(0.0221)		
*HHI*		0.0917 *	
		(0.0479)	
*EU_adj*			−0.00721 *
			(0.00418)
Size	0.0499 ***	0.0475 ***	0.0483 ***
	(0.0102)	(0.0101)	(0.0102)
*TobinQ*	−0.00672	−0.00683	−0.00679
	(0.00535)	(0.00536)	(0.00536)
*ROA*	−0.131	−0.133	−0.131
	(0.0862)	(0.0862)	(0.0862)
*Lev*	0.0955 **	0.0938 **	0.0975 **
	(0.0415)	(0.0415)	(0.0415)
Largest	−0.00344 ***	−0.00355 ***	−0.00348 ***
	(0.000617)	(0.000616)	(0.000618)
Dual	−0.0383 ***	−0.0381 ***	−0.0386 ***
	(0.0142)	(0.0142)	(0.0142)
*CGB*	−0.188	−0.179	−0.176
	(0.130)	(0.130)	(0.130)
Dud	0.00119	0.00122	0.00116
	(0.00107)	(0.00107)	(0.00107)
Constant	−0.829 ***	−0.773 ***	−0.795 ***
	(0.218)	(0.217)	(0.218)
Observation	17,924	17,924	17,924
Adjusted R^2^	0.056	0.056	0.056
Industry, Year	Control		

*t*-statistics in parentheses. *** *p* < 0.01, ** *p* < 0.05, * *p* < 0.1.

**Table 12:** Regression Analysis of Environmental Uncertainty and Enterprises’ Green Technological Innovation Lagging Two Periods.

Variable	(1)	(2)	(3)
	L2.ln*GI*	L2.ln*GI*	L2.ln*GI*
*EU*	0.0771 ***		
	(0.0236)		
*HHI*		0.0866 *	
		(0.0515)	
*EU_adj*			−0.00986 **
			(0.00447)
Size	0.0366 ***	0.0338 ***	0.0354 ***
	(0.0111)	(0.0111)	(0.0111)
*TobinQ*	−0.00894	−0.00894	−0.00893
	(0.00569)	(0.00569)	(0.00569)
*ROA*	−0.216 **	−0.217 **	−0.214 **
	(0.0915)	(0.0915)	(0.0915)
*Lev*	0.0791 *	0.0786 *	0.0815 *
	(0.0448)	(0.0449)	(0.0448)
Largest	−0.00283 ***	−0.00296 ***	−0.00283 ***
	(0.000665)	(0.000664)	(0.000667)
Dual	−0.0454 ***	−0.0451 ***	−0.0461 ***
	(0.0150)	(0.0150)	(0.0150)
*CGB*	−0.139	−0.129	−0.125
	(0.161)	(0.161)	(0.161)
Dud	0.000829	0.000890	0.000812
	(0.00114)	(0.00114)	(0.00114)
Constant	−0.548 **	−0.485 **	−0.522 **
	(0.241)	(0.241)	(0.241)
Observation	15,607	15,607	15,607
Adjusted R^2^	0.054	0.053	0.053
Industry, Year	Control		

*t*-statistics in parentheses. *** *p* < 0.01, ** *p* < 0.05, * *p* < 0.1.

**Table 13:** Regression Analysis of Environmental Uncertainty, Environmental Regulation and Enterprises’ Green Technological Innovation Lagging One Period.

Variable	(1)	(2)	(3)	(4)
	L.ln*GI*	L.ln*GI*	L.ln*GI*	L.ln*GI*
*ER*	0.0695 ***	0.0579 ***	0.0869 ***	0.0575 ***
	(0.0213)	(0.00973)	(0.0104)	(0.00973)
*EU*		0.180 ***		
		(0.0233)		
*EU* × *ER*		0.144 ***		
		(0.0344)		
*HHI*			0.232 ***	
			(0.0426)	
*HHI* × *ER*			0.443 ***	
			(0.0803)	
*EU_adj*				−0.0386 ***
				(0.00425)
*EU_adj* × *ER*				−0.0369 ***
				(0.00776)
Size	0.0463 ***	0.192 ***	0.190 ***	0.192 ***
	(0.0101)	(0.00772)	(0.00838)	(0.00772)
*TobinQ*	−0.00668	0.0282 ***	0.0458 ***	0.0287 ***
	(0.00536)	(0.00522)	(0.00535)	(0.00521)
*ROA*	−0.133	0.230 **	−0.143	0.196 **
	(0.0862)	(0.0984)	(0.102)	(0.0980)
*Lev*	0.0932 **	0.0136	−0.0410	0.0147
	(0.0415)	(0.0347)	(0.0351)	(0.0347)
Largest	−0.00352 ***	−0.00105 ***	−0.00179 ***	−0.00101 **
	(0.000616)	(0.000394)	(0.000433)	(0.000394)
Dual	−0.0373 ***	−0.00441	0.0181	−0.00557
	(0.0142)	(0.0144)	(0.0155)	(0.0144)
*CGB*	−0.160	0.100	0.692 ***	0.0923
	(0.130)	(0.0721)	(0.0729)	(0.0719)
Dud	0.00125	0.000550	−0.000168	0.000695
	(0.00107)	(0.00107)	(0.00115)	(0.00107)
Constant	−0.812 ***	−4.212 ***	−4.006 ***	−4.217 ***
	(0.217)	(0.168)	(0.180)	(0.168)
Observation	17,924	17,924	17,924	17,924
Adjusted R^2^	0.056	0.248	0.095	0.249
Industry, Year	Control			

*t*-statistics in parentheses. *** *p* < 0.01, ** *p* < 0.05.

**Table 14:** The Regression Analysis of Environmental Uncertainty, Environmental Regulation and Enterprises’ Green Technological Innovation Lagging Two Periods.

Variable	(1)	(2)	(3)	(4)
	L2.ln*GI*	L2.ln*GI*	L2.ln*GI*	L2.ln*GI*
*ER*	0.0498 ***	0.0465 ***	0.0750 ***	0.0453 ***
	(0.0102)	(0.0101)	(0.0108)	(0.0102)
*EU*		0.177 ***		
		(0.0241)		
*EU* × *ER*		0.116 ***		
		(0.0339)		
*HHI*			0.233 ***	
			(0.0445)	
*HHI* × *ER*			0.444 ***	
			(0.0835)	
*EU_adj*				−0.0371 ***
				(0.00453)
*EU_adj* × *ER*				−0.0343 ***
				(0.00825)
Size	0.183 ***	0.181 ***	0.182 ***	0.181 ***
	(0.00803)	(0.00803)	(0.00873)	(0.00803)
*TobinQ*	0.0248 ***	0.0255 ***	0.0434 ***	0.0258 ***
	(0.00523)	(0.00523)	(0.00539)	(0.00524)
*ROA*	0.137	0.112	−0.310 ***	0.0832
	(0.106)	(0.106)	(0.110)	(0.106)
*Lev*	−0.00547	0.00294	−0.0571	0.00384
	(0.0364)	(0.0363)	(0.0363)	(0.0363)
Largest	−0.00111 ***	−0.00106 ***	−0.00182 ***	−0.00102 **
	(0.000409)	(0.000408)	(0.000450)	(0.000408)
Dual	−0.0104	−0.00963	0.0117	−0.0108
	(0.0152)	(0.0151)	(0.0164)	(0.0151)
*CGB*	0.0593	0.0617	0.666 ***	0.0572
	(0.0818)	(0.0819)	(0.0834)	(0.0817)
Dud	0.000617	0.000561	−7.36 × 10^-5^	0.000683
	(0.00112)	(0.00112)	(0.00121)	(0.00112)
Constant	−4.077 ***	−3.992 ***	−3.879 ***	−3.998 ***
	(0.178)	(0.178)	(0.192)	(0.178)
Observation	15,607	15,607	15,607	15,607
Adjusted R^2^	0.242	0.244	0.092	0.245
Industry, Year	Control			

*t*-statistics in parentheses. *** *p* < 0.01, ** *p* < 0.05.

## Data Availability

The data of green technological innovation are from the State Intellectual Property Office. The environmental regulation data are mainly from China Environmental Statistics Yearbook; Other research data are mainly from CSMAR database. The data will be made available on request from the corresponding author.

## Appendix A

**Table A1:** Robust Analysis of Environmental Uncertainty and Enterprises’ Green Technological Innovation.

Variable	(1)	(2)	(3)
	ln*GI*1	ln*GI*1	ln*GI*1
*EU*	0.204 ***		
	(0.0153)		
*HHI*		0.212 ***	
		(0.0335)	
*EU_adj*			−0.0398 ***
			(0.00358)
Size	0.143 ***	0.145 ***	0.140 ***
	(0.00720)	(0.00719)	(0.00719)
*TobinQ*	0.0379 ***	0.0370 ***	0.0381 ***
	(0.00464)	(0.00464)	(0.00465)
*ROA*	0.117	0.148 *	0.118
	(0.0856)	(0.0858)	(0.0859)
*Lev*	0.0346	0.0237	0.0432
	(0.0295)	(0.0295)	(0.0295)
Largest	−0.000966 ***	−0.00105 ***	−0.00102 ***
	(0.000364)	(0.000364)	(0.000364)
Dual	0.0138	0.0132	0.0149
	(0.0130)	(0.0131)	(0.0130)
*CGB*	0.657 ***	0.659 ***	0.661 ***
	(0.0576)	(0.0576)	(0.0574)
Dud	−0.00119	−0.00119	−0.00110
	(0.000994)	(0.000996)	(0.000994)
Constant	−2.926 ***	−2.961 ***	−2.858 ***
	(0.154)	(0.154)	(0.153)
Observation	21,359	21,359	21,359
Adjusted R^2^	0.072	0.069	0.071
Industry, Year	Control		

*t*-statistics in parentheses. *** *p* < 0.01, * *p* < 0.1.

**Table A2:** Robustness Analysis of Environmental Uncertainty, Environmental Regulation and Enterprises’ Green Technological Innovation.

Variable	(1)	(2)	(3)	(4)
	ln*GI*1	ln*GI*1	ln*GI*1	ln*GI*1
*ER*	0.0763 ***	0.0725 ***	0.0735 ***	0.0740 ***
	(0.00878)	(0.00873)	(0.00874)	(0.00874)
*EU*		0.208 ***		
		(0.0158)		
*EU* × *ER*		0.118 ***		
		(0.0287)		
*HHI*			0.222 ***	
			(0.0334)	
*HHI* × *ER*			0.241 ***	
			(0.0629)	
*EU_adj*				−0.0399 ***
				(0.00370)
*EU_adj* × *ER*				−0.0154 **
				(0.00681)
Size	0.146 ***	0.145 ***	0.146 ***	0.143 ***
	(0.00720)	(0.00720)	(0.00717)	(0.00719)
*TobinQ*	0.0391 ***	0.0403 ***	0.0391 ***	0.0406 ***
	(0.00467)	(0.00465)	(0.00465)	(0.00466)
*ROA*	0.132	0.0888	0.122	0.0835
	(0.0861)	(0.0855)	(0.0857)	(0.0857)
*Lev*	0.0235	0.0310	0.0203	0.0386
	(0.0295)	(0.0295)	(0.0295)	(0.0295)
Largest	−0.00103 ***	−0.000843 **	−0.000934 **	−0.000903 **
	(0.000364)	(0.000363)	(0.000363)	(0.000364)
Dual	0.0125	0.0118	0.0125	0.0125
	(0.0130)	(0.0130)	(0.0130)	(0.0130)
*CGB*	0.644 ***	0.635 ***	0.634 ***	0.638 ***
	(0.0576)	(0.0576)	(0.0577)	(0.0575)
Dud	−0.000831	−0.000932	−0.000926	−0.000780
	(0.000995)	(0.000993)	(0.000995)	(0.000992)
Constant	−3.076 ***	−3.034 ***	−3.053 ***	−2.981 ***
	(0.154)	(0.154)	(0.154)	(0.154)
Observation	21,359	21,359	21,359	21,359
Adjusted R^2^	0.071	0.076	0.073	0.075
Industry, Year	Control			

*t*-statistics in parentheses. *** *p* < 0.01, ** *p* < 0.05.

**Table A3:** Robustness Analysis of Environmental Uncertainty and Enterprises’ Green Technological Innovation.

Variable	(1)	(2)	(3)
	ln*GI*	ln*GI*	ln*GI*
*EU*1	0.636 ***		
	(0.157)		
*HHI*1		0.128 **	
		(0.0538)	
*EU_unadj*			−0.0966 ***
			(0.0325)
Size	0.0526 ***	0.0507 ***	0.0518 ***
	(0.00899)	(0.00898)	(0.00900)
*TobinQ*	−0.00851 *	−0.00839 *	−0.00848 *
	(0.00478)	(0.00478)	(0.00478)
*ROA*	−0.0165	−0.0200	−0.0105
	(0.0779)	(0.0779)	(0.0780)
*Lev*	0.0609*	0.0574	0.0635 *
	(0.0367)	(0.0367)	(0.0367)
Largest	−0.00359 ***	−0.00369 ***	−0.00356 ***
	(0.000539)	(0.000538)	(0.000540)
Dual	−0.0245 *	−0.0242 *	−0.0251 *
	(0.0129)	(0.0129)	(0.0129)
*CGB*	0.0586	0.0700	0.0605
	(0.104)	(0.104)	(0.105)
Dud	0.00139	0.00141	0.00136
	(0.000987)	(0.000988)	(0.000988)
Constant	−0.838 ***	−0.790 ***	−0.819 ***
	(0.192)	(0.191)	(0.192)
Observation	21,359	21,359	21,359
Adjusted R^2^	0.058	0.058	0.058
Industry, Year	Control		

*t*-statistics in parentheses. *** *p* < 0.01, ** *p* < 0.05, * *p* < 0.1.

**Table A4:** Robustness Analysis of Environmental Regulation, Environmental Uncertainty and Enterprises’ Green Technological Innovation.

Variable	(1)	(2)	(3)
	ln*GI*	ln*GI*	ln*GI*
*ER*	0.0791 ***	0.0795 ***	0.0799 ***
	(0.00953)	(0.00953)	(0.00955)
*EU*1	1.862 ***		
	(0.151)		
*EU*1 × *ER*	1.433 ***		
	(0.281)		
*HHI*1		0.306 ***	
		(0.0439)	
*HHI*1 × *ER*		0.500 ***	
		(0.0823)	
*EU_unadj*			−0.419 ***
			(0.0314)
*EU_unadj* × *ER*			−0.142 **
			(0.0553)
Size	0.194 ***	0.194 ***	0.191 ***
	(0.00790)	(0.00785)	(0.00789)
*TobinQ*	0.0444 ***	0.0435 ***	0.0452 ***
	(0.00499)	(0.00498)	(0.00500)
*ROA*	0.0659	0.0978	0.0463
	(0.0940)	(0.0941)	(0.0941)
*Lev*	−0.0243	−0.0380	−0.00576
	(0.0324)	(0.0324)	(0.0324)
Largest	−0.00139 ***	−0.00146 ***	−0.00138 ***
	(0.000400)	(0.000399)	(0.000400)
Dual	0.0283 **	0.0296 **	0.0293 **
	(0.0143)	(0.0144)	(0.0143)
*CGB*	0.701 ***	0.701 ***	0.711 ***
	(0.0616)	(0.0618)	(0.0615)
Dud	−9.43 × 10^−5^	−0.000173	0.000247
	(0.00110)	(0.00110)	(0.00110)
Constant	−4.065 ***	−4.061 ***	−4.003 ***
	(0.169)	(0.168)	(0.169)
Observation	21,359	21,359	21,359
Adjusted R^2^	0.099	0.097	0.100
Industry, Year	Control		

*t*-statistics in parentheses. *** *p* < 0.01, ** *p* < 0.05.

**Table A5:** Robustness Analysis of Environmental Uncertainty and Enterprises’ Green Technological Innovation Based on Poisson Regression.

Variable	(1)	(2)	(3)
	*GI*	*GI*	*GI*
*EU*	0.154 ***		
	(0.0308)		
*HHI*		1.090 ***	
		(0.0537)	
*EU_adj*			−0.0298 ***
			(0.00741)
Size	1.132 ***	1.145 ***	1.131 ***
	(0.0107)	(0.0107)	(0.0107)
*TobinQ*	0.114 ***	0.129 ***	0.114 ***
	(0.00796)	(0.00796)	(0.00796)
*ROA*	−1.836 ***	−1.967 ***	−1.833 ***
	(0.138)	(0.138)	(0.138)
*Lev*	−2.325 ***	−2.370 ***	−2.308 ***
	(0.0665)	(0.0665)	(0.0664)
Largest	−0.00913 ***	−0.00818 ***	−0.00877 ***
	(0.000911)	(0.000912)	(0.000916)
Dual	0.231 ***	0.191 ***	0.238 ***
	(0.0167)	(0.0167)	(0.0167)
*CGB*	0.287	0.341 *	0.261
	(0.175)	(0.174)	(0.175)
Dud	0.0129 ***	0.00985 ***	0.0129 ***
	(0.00115)	(0.00116)	(0.00115)
Constant	−24.02 ***	−24.11 ***	−24.01 ***
	(0.252)	(0.252)	(0.252)
Observation	21,359	21,359	21,359
Industry, Year	Control		

*t*-statistics in parentheses. *** *p* < 0.01, * *p* < 0.1.

**Table A6:** Robustness Analysis of Environmental Regulation, Environmental Uncertainty and Enterprises’ Green Technological Innovation Based on Poisson Regression.

Variable	(1)	(2)	(3)	(4)
	*GI*	*GI*	*GI*	*GI*
*ER*	0.158 ***	0.120 ***	0.250 ***	0.134 ***
	(0.0212)	(0.0216)	(0.00631)	(0.00640)
*EU*		0.407 ***		
		(0.0415)		
*EU* × *ER*		0.596 ***		
		(0.0617)		
*HHI*			0.143 ***	
			(0.0304)	
*HHI* × *ER*			2.224 ***	
			(0.0448)	
*EU_adj*				−0.185 ***
				(0.00487)
*EU_adj* × *ER*				0.163 ***
				(0.00702)
Size	1.125 ***	1.126 ***	1.078 ***	1.262 ***
	(0.0107)	(0.0106)	(0.00297)	(0.00361)
*TobinQ*	0.118 ***	0.121 ***	0.0595 ***	0.124 ***
	(0.00795)	(0.00796)	(0.00191)	(0.00459)
*ROA*	−1.818 ***	−1.867 ***	2.450 ***	0.321 ***
	(0.138)	(0.138)	(0.0894)	(0.102)
*Lev*	−2.296 ***	−2.294 ***	−1.591 ***	−2.283 ***
	(0.0664)	(0.0665)	(0.0274)	(0.0273)
Largest	−0.00866 ***	−0.00827 ***	−0.0157 ***	0.00112 ***
	(0.000910)	(0.000913)	(0.000261)	(0.000247)
Dual	0.241 ***	0.245 ***	0.858 ***	0.853 ***
	(0.0167)	(0.0167)	(0.00919)	(0.00918)
*CGB*	0.331 *	0.374 **	2.076 ***	2.523 ***
	(0.175)	(0.175)	(0.0443)	(0.0446)
Dud	0.0137 ***	0.0136 ***	−0.0253 ***	−0.0354 ***
	(0.00115)	(0.00115)	(0.000659)	(0.000714)
Constant	−24.09 ***	−24.05 ***	−22.43 ***	−26.28 ***
	(0.251)	(0.251)	(0.0659)	(0.0832)
Observation	21,359	21,359	21,359	21,359
Industry, Year	Control			

*t*-statistics in parentheses. *** *p* < 0.01, ** *p* < 0.05, * *p* < 0.1.
